# Supplementary figures and images for: Association between optical coherence tomography-defined culprit morphologies and changes in hyperemic coronary flow after elective stenting assessed by transthoracic Doppler echocardiography
Source: PLoS One. 2024 Aug 15;19(8):e0307384. doi: 10.1371/journal.pone.0307384 (PMC11326549; doi:10.1371/journal.pone.0307384)

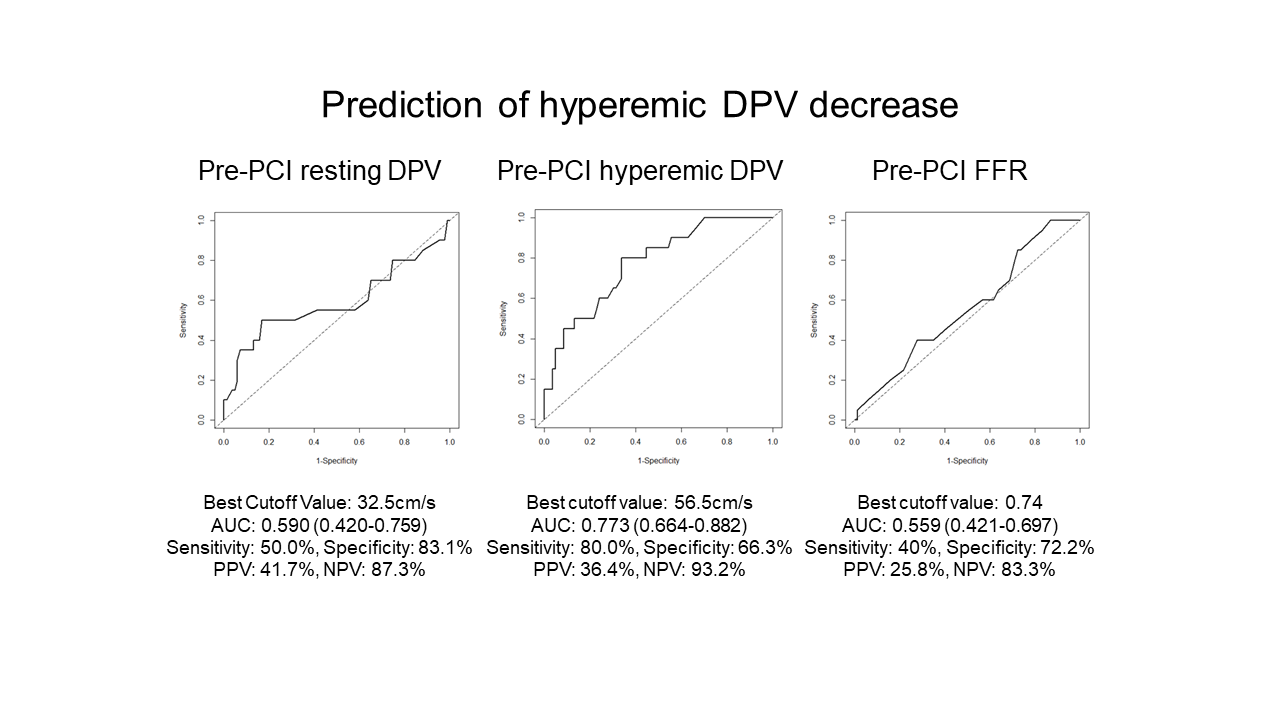

Supplement: S1 Fig — Receiver-operating characteristic curve analysis to predict %hDPV decrease. AUC, area under the curve; CI, confidence interval; DPV, diastolic peak velocity; FFR, fractional flow reserve; PCI, percutaneous coronary intervention. (TIF) [file pone.0307384.s002.tif]

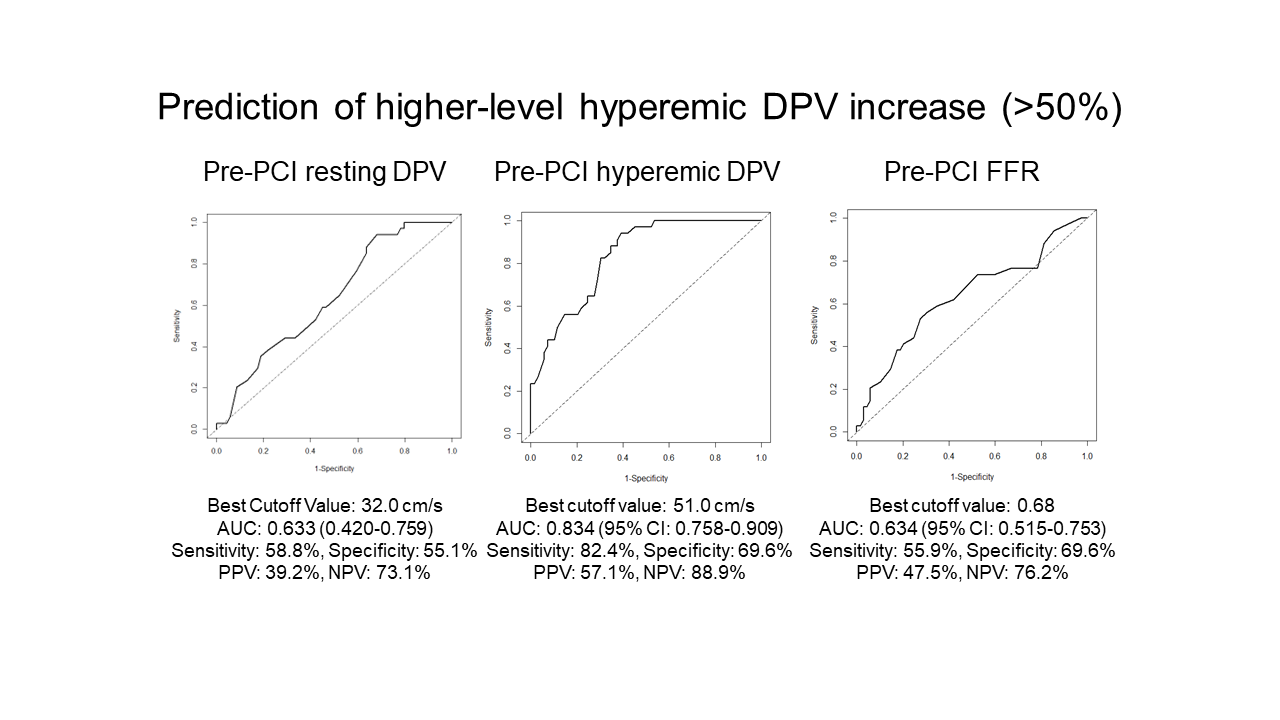

Supplement: S2 Fig — Receiver-operating characteristic curve analysis to predict higher-level %hDPV increase (>50%). AUC, area under the curve; CI, confidence interval; DPV, diastolic peak velocity; FFR, fractional flow reserve; PCI, percutaneous coronary intervention. (TIF) [file pone.0307384.s003.tif]
