# Supplementary material for: Association between optical coherence tomography-defined culprit morphologies and changes in hyperemic coronary flow after elective stenting assessed by transthoracic Doppler echocardiography
Source: PLoS One. 2024 Aug 15;19(8):e0307384. doi: 10.1371/journal.pone.0307384 (PMC11326549; doi:10.1371/journal.pone.0307384)
Supplement: S1 Table — Values are reported as n (%) or the median (25-75th percentile). DPV, diastolic peak velocity; HDL, high-density lipoprotein; LDL, low-density lipoprotein; PCI, percutaneous coronary intervention. (DOCX) [file pone.0307384.s004.docx]

**S1 Table. Clinical demographics and angiographic findings according to the presence or absence of layered plaque in the vessel**

|  | Total  (n=103) | Presence of layered plaque  (n = 59) | Absence of layered plaque  (n = 44) | P-value |
| --- | --- | --- | --- | --- |
| Clinical demographics | | | | |
| Age, years | 72.0 [60.0, 76.5] | 71.0 [58.5, 75.0] | 73.0 [65.0, 77.3] | 0.10 |
| Male gender | 87 (84.5) | 52 (88.1) | 35 (79.5) | 0.36 |
| Prior myocardial infarction | 32 (31.1) | 20 (33.9) | 12 (27.3) | 0.62 |
| Hypertension | 80 (77.7) | 41 (69.5) | 39 (88.6) | 0.04 |
| Dyslipidemia | 63 (61.2) | 35 (59.3) | 28 (63.6) | 0.81 |
| Diabetes Mellitus | 41 (39.8) | 23 (39.0) | 18 (40.9) | 1.00 |
| Current smoking | 27 (26.2) | 16 (27.1) | 11 (25.0) | 0.99 |
| C-reactive protein, mg/dL | 0.06 [0.03, 0.17] | 0.07 [0.03, 0.15] | 0.04 [0.03, 0.17] | 0.41 |
| Estimated glomerular filtration rate, ml/min/1.73m^2^ | 66.8 [54.1, 76.8] | 66.5 [53.2, 74.6] | 67.4 [54.7, 78.8] | 0.36 |
| Total cholesterol, mg/dL | 152.0 [125.5, 176.0] | 152.0 [126.5, 174.0] | 153.5 [126.3, 177.0] | 0.97 |
| LDL cholesterol, mg/dL | 75.0 [56.0, 95.0] | 73.0 [55.0, 97.5] | 76.0 [58.5, 94.0] | 0.88 |
| HDL cholesterol, mg/dL | 54.0 [44.3, 65.0] | 53.0 [42.3, 59.0] | 58.0 [47.5, 67.3] | 0.06 |
| Triglycerides, mg/dL | 103.0 [73.0, 148.0] | 120.0 [87.5, 193.0] | 89.0 [63.8, 136.3] | <0.01 |
| Post-PCI creatine kinase myocardial band, IU/L | 7.0 [4.0, 12.5] | 7.0 [4.0, 12.5] | 6.0 [4.0, 12.5] | 0.54 |
| Post-PCI high-sense troponin I, ng/L | 404.0 [173.0, 1284.5] | 398.0 [167.0, 1457.5] | 404.0 [199.3, 830.8] | 0.96 |
| Left ventricular ejection fraction, % | 65.0 [59.5, 70.0] | 63.0 [56.0, 69.0] | 66.0 [61.0, 71.0] | 0.053 |
| Angiographic findings | | | | |
| Minimal lumen diameter, mm | 0.90 [0.73, 1.12] | 0.90 [0.72, 1.10] | 0.90 [0.77, 1.18] | 0.49 |
| Reference diameter, mm | 2.52 [2.17, 3.03] | 2.46 [2.14, 2.94] | 2.70 [2.20, 3.11] | 0.38 |
| Diameter stenosis, % | 64.0 [55.8, 70.7] | 63.9 [57.3, 70.1] | 64.1 [55.1, 72.3] | 0.83 |
| Lesion length, mm | 16.3 [11.9, 25.0] | 17.0 [12.2, 25.9] | 15.4 [11.3, 22.3] | 0.20 |

Values are reported as n (%) or the median (25-75th percentile). DPV, diastolic peak velocity; HDL, high-density lipoprotein; LDL, low-density lipoprotein; PCI, percutaneous coronary intervention.
