# Supplementary material for: Association between optical coherence tomography-defined culprit morphologies and changes in hyperemic coronary flow after elective stenting assessed by transthoracic Doppler echocardiography
Source: PLoS One. 2024 Aug 15;19(8):e0307384. doi: 10.1371/journal.pone.0307384 (PMC11326549; doi:10.1371/journal.pone.0307384)
Supplement: S2 Table — Values are reported as n (%) or the median (interquartile range). CFVR, coronary flow velocity reserve; DPV, diastolic peak velocity; FFR, fractional flow reserve; PCI, percutaneous coronary intervention; Tmn, mean transit time. (DOCX) [file pone.0307384.s005.docx]

**S2 Table. Physiological parameters according to the presence or absence of layered plaque in the vessel**

|  | Total  (n=103) | Presence of layered plaque  (n = 59) | Absence of layered plaque  (n = 44) | P-value |
| --- | --- | --- | --- | --- |
| Pre PCI FFR | 0.71 [0.65, 0.74] | 0.71 [0.64, 0.74] | 0.70 [0.64, 0.74] | 0.91 |
| Pre-PCI resting DPV, cm/s | 27.0 [21.0, 32.0] | 27.0 [20.0, 33.0] | 27.0 [22.0, 30.5] | 0.82 |
| Pre-PCI hyperemic DPV, cm/s | 52.0 [42.0, 66.0] | 53.0 [42.5, 65.5] | 51.0 [39.8, 67.0] | 0.51 |
| Pre-PCI CFVR | 2.00 [1.60, 2.37] | 2.00 [1.63, 2.46] | 1.98 [1.49, 2.17] | 0.29 |
| Hyperemic DPV increase, % | 27.3 [6.32, 59.1] | 16.7 [-3.3, 55.1] | 39.8 [17.4, 63.6] | 0.02 |

Values are reported as n (%) or the median (interquartile range). CFVR, coronary flow velocity reserve; DPV, diastolic peak velocity; FFR, fractional flow reserve; PCI, percutaneous coronary intervention; T_mn_, mean transit time.
