# Supplementary material for: Association between optical coherence tomography-defined culprit morphologies and changes in hyperemic coronary flow after elective stenting assessed by transthoracic Doppler echocardiography
Source: PLoS One. 2024 Aug 15;19(8):e0307384. doi: 10.1371/journal.pone.0307384 (PMC11326549; doi:10.1371/journal.pone.0307384)
Supplement: S3 Table — Values are reported as n (%) or the median (25-75th percentile). (DOCX) [file pone.0307384.s006.docx]

**S3 Table. Optical coherence tomographic findings according to the presence or absence of layered plaque in the vessel**

|  | Total  (n=103) | Presence of layered plaque  (n = 59) | Absence of layered plaque  (n = 44) | P-value |
| --- | --- | --- | --- | --- |
| Minimal lumen area, mm^2^ | 1.34 [1.05, 1.87] | 1.29 [1.09, 1.69] | 1.44 [1.03, 1.94] | 0.37 |
| Thin-cap fibroatheroma | 14 (13.6) | 11 (18.6) | 3 (6.8) | 0.15 |
| Plaque rupture | 20 (19.4) | 14 (23.7) | 7 (15.9) | 0.47 |
| Lipid-rich plaque | 70 (68.0) | 46 (78.0) | 24 (54.5) | 0.02 |
| Maximum lipid angle, degree | 120.6 [0.0, 183.2] | 125.9 [91.5, 200.2] | 103.6 [0.0, 145.8] | 0.04 |
| Fibrous cap thickness, µm | 120 [90, 225] | 0.10 [0.06, 0.13] | 0.11 [0.00, 0.23] | 0.53 |
| Lipid length, mm | 5.4 [0.0, 9.6] | 6.1 [3.9, 11.8] | 4.9 [0.0, 9.2] | 0.11 |
| Microchannel | 33 (32.0) | 20 (33.9) | 13 (29.5) | 0.80 |
| Macrophage | 85 (82.5) | 52 (88.1) | 33 (75.0) | 0.14 |
| Cholesterol crystal | 41 (39.8) | 25 (42.4) | 16 (36.4) | 0.68 |
| Calcification | 75 (72.8) | 42 (71.2) | 33 (75.0) | 0.84 |

Values are reported as n (%) or the median (25-75th percentile).
